# Cross-Modal Interaction Fusion-Based Uncertainty-Aware Prediction Method for Industrial Froth Flotation Concentrate Grade by Using a Hybrid SKNet-ViT Framework

**DOI:** 10.3390/s26010150

**Published:** 2025-12-25

**Authors:** Fanlei Lu, Weihua Gui, Yulong Wang, Jiayi Zhou, Xiaoli Wang

**Affiliations:** School of Automation, Central South University, Changsha 410083, China

**Keywords:** flotation grade prediction, cross-modal feature fusion, quantile regression, localized conformal prediction, uncertainty estimation

## Abstract

In froth flotation, the features of froth images are important information to predict the concentrate grade. However, the froth structure is influenced by multiple factors, such as air flowrate, slurry level, ore properties, reagents, etc., which leads to highly complex and dynamic changes in the image features. Additionally, issues such as the immeasurability of ore properties and measurement errors pose significant uncertainties including aleatoric uncertainty (intrinsic variability from ore fluctuations and sensor noise) and epistemic uncertainty (incomplete feature representation and local data heterogeneity) and generalization challenges for prediction models. This paper proposes an uncertainty quantification regression framework based on cross-modal interaction fusion, which integrates the complementary advantages of Selective Kernel Networks (SKNet) and Vision Transformers (ViT). By designing a cross-modal interaction module, the method achieves deep fusion of local and global features, reducing epistemic uncertainty caused by incomplete feature expression in single-models. Meanwhile, by combining adaptive calibrated quantile regression—using exponential moving average (EMA) to track real-time coverage and adjust parameters dynamically—the prediction interval coverage is optimized, addressing the inability of static quantile regression to adapt to aleatoric uncertainty. And through the localized conformal prediction module, sensitivity to local data distributions is enhanced, avoiding the limitation of global conformal methods in ignoring local heterogeneity. Experimental results demonstrate that this method significantly improves the robustness of uncertainty estimation while maintaining high prediction accuracy, providing strong support for intelligent optimization and decision-making in industrial flotation processes.

## 1. Introduction

Froth flotation is a critical process in mineral processing, which separates valuable minerals from gangue based on their physicochemical properties. Accurate and timely measurement of concentrate grade is paramount for optimization and control of the flotation process to enhance resource utilization [1]. However, complex on-site environment, high cost, and maintenance demand of the online analysis instruments mean that the industrial operations often rely on manual observation or offline laboratory analysis (e.g., X-ray fluorescence). These methods are subjective and introduce significant time lags (typically 1–2 h), which is inadequate for real-time closed-loop control [2]. Since froth flotation images contain a wealth of process state information, image-based grade soft sensing methods have become an active research focus.

Meanwhile, with the rapid development of deep learning and cumulative of process data, automatic feature extraction and representation methods, and prediction methods based on deep neural networks for flotation grade have been researched broadly [3]. Methodologically, most works rely on Convolutional Neural Networks (CNNs) because froth images carry rich cues. Wang et al. [4] proposed a two-step operating condition recognition strategy based on image sequences, where deep learning was used to classify the images to obtain the conditions of antimony flotation, and satisfactory classification results were achieved. Fu et al. [5,6] investigated several popular CNNs for classification of arsenic flotation conditions, and experiment results showed that the ResNet achieved the best classification results. Zarie et al. [7] developed a froth image classification method based on CNN for process monitoring. The study demonstrated that the CNN classifier effectively extracts froth features and its classification accuracy outperforms traditional artificial neural networks (ANNs). Jia et al. [8] proposed a CNN-based performance evaluation method with feature learning and expert feedback fusion, which improves robustness and accuracy under image distortions. Beyond purely spatial cues, sequence models capture temporal dynamics. Zhou et al. [9] proposed a domain-knowledge-embedded deep Long Short-Term Memory (LSTM) soft measurement framework (DKST-DLSTM), which significantly improved the prediction accuracy of key indicators such as concentrate grade by extracting spatio-temporal features in the flotation process. Pu et al. [10] used LSTM to predict iron and silicon waste concentrations by using industrial data, which outperforms random forests, and Zhang et al. [11] combined feed grade refined embedding (FGRE) and nonoverlapping patch encoding (NOPE) with RNNs to enhance dynamic monitoring accuracy. In addition, graph-based and attention models have been explored too. Wang et al. [12] proposed a sparse-attention spectral graph neural network (SASGNN) for state-variable prediction, while Lu et al. [13] developed a multimode performance assessment method using a deep embedded graph clustering network that integrates froth images and process variables via Bayesian fusion. Complementary to these architectures, Xie et al. [14] employed an mRMR plus multiswarm PSO-based sensitive feature selection with collaborative search, coupled with a self-adjusting RBF neural network, to further improve flotation prediction accuracy. Despite progress in deep learning for flotation grade prediction, most methods provide only point estimates without calibrated uncertainty. Uncertainty arises from two sources: aleatoric (intrinsic process variability such as changing ore conditions, operating fluctuations, and sensor noise) and epistemic (limited/delayed labels, unobserved variables, and covariate shift). Hence, soft sensors should output accurate predictions with reliable intervals under distributional change. While CNNs emphasize local details [15] and ViTs capture global context [16], a single model struggles to account for both local variability and global trends.

For modeling uncertainty in industrial processes, primary approaches include probabilistic distribution modeling [17], quantile regression [18], and conformal prediction [19]. While these methods provide interval estimates, they largely depend on global statistical characteristics, making it difficult to adapt in real-time to local distributional variations caused by raw material or process fluctuations in flotation operations. Moreover, static calibration may result in prediction intervals being either too wide or too narrow [20], thereby affecting reliability. Although research on uncertainty prediction for flotation processes is scarce, advanced techniques have emerged in other complex systems. For instance, in probabilistic distribution prediction, Dong et al. [21] proposed an uncertainty-guided graph neural network for air quality forecasting, Sun et al. [22] developed a multi-distribution ensemble framework, and Trapero et al. [23] combined kernel density estimation with forecast errors. In terms of hybrid modeling and interval estimation, Wang et al. [24] employed an integrated Empirical Mode Decomposition (EMD) and Relevance Vector Machine (RVM) model for photovoltaic power output, Khosravi et al. [25] proposed a neural network-based method for constructing prediction intervals, and Hou et al. [26] developed an uncertainty-aware deep learning framework for risk identification. Other methods, such as the non-parametric quantile approach by Bremnes et al. [27] or the fuzzy theory-based intervals by Pierre et al. [28], are often limited by linear structure assumptions or reliance on expert knowledge, respectively. Despite these advances, their direct application is challenged by the unique multi-modal data and dynamic, nonlinear behavior of flotation—even as effective multi-modal fusion strategies have been developed for other scenarios [29,30]. Therefore, how to integrate multi-modal deep features and achieve dynamic, locally sensitive uncertainty quantification remains an urgent and unresolved challenge.

Although intelligent methods have been extensively explored for uncertainty modeling in industrial processes [31], they often suffer from insufficient robustness and limited generalization capability when they face high-noise and highly dynamic of the input data, making it difficult to meet the practical demand for high-confidence interval prediction in production. Therefore, integrating multi-modal structures with stronger expressive power and theoretically guaranteed out-of-distribution uncertainty calibration has become a key breakthrough in current research.

In addition, traditional quantile regression and global conformal prediction methods also exhibit significant limitations in constructing prediction intervals. On one hand, these methods typically rely on the statistical characteristics of global data, making it difficult to adapt in real-time to local distributional differences in flotation processes caused by variations in ore properties or fluctuations of operating conditions. On the other hand, static calibration methods often produce prediction intervals that are either too wide or too narrow in the face of dynamically changing data distributions, thereby reducing the reliability of decision-making in practical applications.

To address the aforementioned challenges, this paper proposes a cross-modal interactive fusion-based uncertainty quantification regression framework for high-precision prediction and uncertainty quantification of concentrate grade in flotation. First, a hybrid architecture integrating SKNet and ViT is constructed, in which bidirectional information interaction is achieved through a cross-attention mechanism, so that local details and global features are effectively combined. Subsequently, we design an adaptive calibrated quantile regression method that introduces an exponential moving average (EMA) update strategy for coverage rate and an adaptive loss function, thereby enabling dynamic adjustment of calibration parameters and enhancing the coverage capability of prediction intervals under dynamic operating conditions. Finally, a conformal prediction module based on local data distribution is developed, which improves the model’s responsiveness to local data fluctuations and further enhances the reliability of prediction intervals by incorporating local neighborhood analysis and nonlinear interval width adjustment.

The main contributions of this paper can be summarized as follows:

(1) A novel cross-modal feature fusion architecture is proposed, which is based on the integration of SKNet (convolutional features) and ViT (global structure) with cross-attention. By designing a cross-modal interaction module, complementary learning between different feature extractors is achieved. This approach enables simultaneous capture of local details and global information in flotation process data, significantly enhancing the model’s capacity to represent complex data patterns.
(2) An adaptive calibrated quantile regression framework is developed, coupling an EMA-driven coverage-rate updater with a loss comprising quantile error and a calibration-penalty term, enabling real-time reweighting and improved interval coverage under nonstationary conditions.
(3) A localized conformal prediction method based on local neighborhood analysis is proposed. By mining the local features of the calibration set and adjusting the interval width nonlinearly, the prediction intervals become highly sensitive to local data distributions. Automatic iterative optimization is used to effectively enhance the reliability of intervals for samples from different regions.
(4) A complete end-to-end framework covering data preprocessing, feature fusion, model training, and uncertainty prediction interval evaluation is constructed. The method’s superior performance on real flotation datasets is systematically validated through multi-dimensional experiments and comprehensive metrics (MAE, MSE, coverage, interval width, etc.), providing technical support for industrial applications.

The structure of this paper is arranged as follows: Section 1 reviews the relevant theoretical foundations and research progress; Section 2 provides a detailed description of the proposed method and its implementation; Section 3 presents experimental design, result analysis, and discussion; and finally, Section 4 concludes the paper and discusses future research directions.

## 2. Methodology: Cross-Modal Modeling Method with Adaptive and Localized Uncertainty

**Figure 1:**
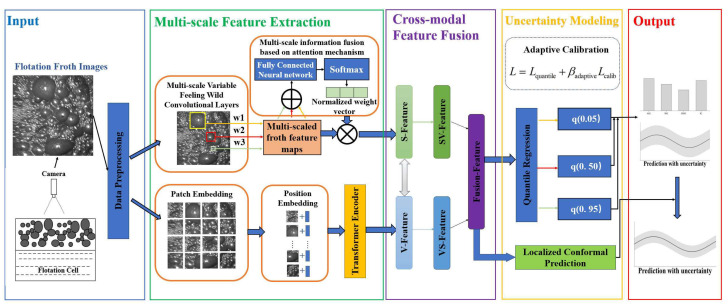
The overall architecture of the proposed cross-modal grade prediction framework.

The system begins by acquiring real-time flotation froth images, which undergo preprocessing to ensure high-quality inputs for subsequent feature extraction. The multi-scale feature extraction module employs both a multi-scale variable receptive field convolutional layer (SKNet) and Vision Transformer (ViT) to extract rich features at different scales. Positional embeddings and a transformer encoder further enhance the representation of spatial structures. In the cross-modal feature fusion stage, the multi-scale features extracted from the images are fused to leverage the correlations between different modalities, thereby enhancing the expressive power of the features. This results in a high-dimensional fused feature vector used for prediction. To address the uncertainty in grade prediction, the system incorporates an uncertainty modeling module. Specifically, we use quantile regression to generate interval predictions. This is combined with localized conformal prediction to quantify the uncertainty of the results while maintaining accuracy. An adaptive calibration mechanism further refines the confidence intervals to align with observed data, improving the model’s reliability in industrial settings. Finally, the system outputs not only the predicted grade value but also the corresponding prediction interval and an analysis of uncertainty, providing a scientific basis for intelligent process control and risk-informed decision-making. The overall architecture of the proposed grade prediction and uncertainty quantification framework is illustrated in Figure 1.

### 2.1. Feature Extraction Backbone: SKNet and ViT

Flotation froth images are characterized by complex local textures and global structural patterns. To simultaneously capture these multi-granular features, we construct a dual-branch backbone comprising a Selective Kernel Network (SKNet) and a Vision Transformer (ViT).

SKNet is employed to extract multi-scale local features. By dynamically adjusting the receptive fields of its convolutional kernels based on the input information, SKNet effectively captures fine-grained details of froth bubbles at varying scales [32]. Complementarily, ViT serves as the global feature extractor. Utilizing the multi-head self-attention mechanism, ViT models long-range dependencies and global semantic contexts across the entire image, compensating for the limited receptive field of traditional CNNs [33].

The detailed mathematical formulations and architectural specifications of both SKNet and ViT are provided in Appendix A.

While these two branches provide rich feature representations independently, simply concatenating them fails to exploit their semantic correlations. To address this, we introduce a cross-modal interaction fusion module in the following section to achieve deep bidirectional information integration.

### 2.2. Fusion of Cross-Modal Features

To fully leverage SKNet’s local detail perception and ViT’s global semantic understanding capabilities, we design a cross-modal interaction fusion module. This module achieves mutual enhancement of features through bidirectional attention mechanisms and generates unified feature representations through deep fusion. Unlike simple concatenation or summation used in standard CNN-transformer hybrids, our proposed cross-modal interaction module (Equations (1)–(4)) employs a bidirectional attention mechanism. This allows the local features from SKNet to modulate the global representations of ViT and vice versa, explicitly modeling the semantic alignment between texture details and global structural patterns.

#### 2.2.1. Bidirectional Attention Modulation

Let the features extracted by SKNet and ViT be $F_{SK}\in R^{d}$ and $F_{ViT}\in R^{d}$, respectively. First, we calculate attention weights of local features on global features:

(1) $$\alpha_{sv}=\sigma \left(W_{sv}\cdot F_{SK}+b_{sv}\right)$$

where $W_{sv}\in R^{d\times d}$ is a learnable projection matrix, and σ(⋅) is the sigmoid activation function.

This attention weight is then used to modulate global features:

(2) $${\hat{F}}_{ViT}=F_{ViT}⊙\alpha_{sv}$$

Similarly, we calculate attention modulation of global features on local features:

(3) $$\alpha_{vs}=\sigma \left(W_{vs}\cdot F_{ViT}+b_{vs}\right)$$

(4) $${\hat{F}}_{SK}=F_{SK}⊙\alpha_{vs}$$

#### 2.2.2. Fusion of Multi-Level Features

Original features and interaction-modulated features are concatenated as,

(5) $$F_{concat}=\left[F_{SK};F_{ViT};{\hat{F}}_{SK};{\hat{F}}_{ViT}\right]\in R^{4d}$$

Deep fusion is performed through multi-layer perceptron (MLP):

(6) Fhidden=ReLU(W1⋅Fconcat+b1)

(7) $$F_{fused}=W_{2}\cdot F_{hidden}+b_{2}$$

where $W_{1}\in R^{2d\times 4d},W_{2}\in R^{d\times 2d}$ are weight matrices.

This fusion strategy preserves unique information from each modality while enhancing feature representation through interaction.

### 2.3. Adaptive Quantile Regression Model

To quantify the uncertainty, we propose an adaptive calibrated quantile regression method which not only predicts multiple quantiles to construct prediction intervals but also ensures interval reliability through dynamic calibration mechanisms.

#### 2.3.1. Quantile Regression Network

For fused features $F_{fused}$, K quantiles are predicted through independent fully connected networks:

(8) $${\hat{y}}_{\tau_{i}}=f_{\tau_{i}}\left(F_{fused};\theta_{\tau_{i}}\right),i=1,2,\ldots ,K$$

where $\tau_{i}\in \left(0,1\right)$ is the target quantile, and $f_{\tau_{i}}\left(\cdot \right)$ is the corresponding prediction network.

#### 2.3.2. Pinball Loss Function

Quantile regression typically employs the Pinball loss function:

(9) $$L_{\tau }\left(y,{\hat{y}}_{\tau }\right)=\{\begin{array}{cc} \tau \left(y-{\hat{y}}_{\tau }\right) & \mathrm{if}\;y\geq {\hat{y}}_{\tau }\\ \left(1-\tau \right)\left({\hat{y}}_{\tau }-y\right) & \mathrm{if}\;y<{\hat{y}}_{\tau } \end{array}$$

The total quantile loss is

(10) $$L_{quantile}=\frac{1}{K}\sum_{i=1}^{K}L_{\tau_{i}}\left(y,{\hat{y}}_{\tau_{i}}\right)$$

#### 2.3.3. Adaptive Calibration Mechanism

To ensure prediction interval coverage aligns with theoretical values, we introduce exponential moving average (EMA) tracking of coverage:

(11) $$C_{EMA}^{\left(t\right)}=\gamma \cdot C_{EMA}^{\left(t-1\right)}+\left(1-\gamma \right)\cdot C_{batch}^{\left(t\right)}$$

where γ=0.9 is the momentum coefficient, and $C_{batch}^{\left(t\right)}$ is the actual coverage of the *t*-th batch.

Target coverage is defined as

(12) $$C_{target,i}=\{\begin{array}{cc} 1-\tau_{i} & \mathrm{if}\;\tau_{i}<0.5\\ \tau_{i} & \mathrm{if}\;\tau_{i}\geq 0.5 \end{array}$$

Calibration loss is defined as

(13) $$L_{calib}=\frac{1}{K}\sum_{i=1}^{K}(C_{EMA,i}-C_{target,i}{)}^{2}$$

Adaptive weight is calculated as

(14) $$\beta_{adaptive}=\beta \cdot \left(1+\lambda \cdot \|C_{EMA}-C_{target}{\|}_{1}\right)$$

where β is the base weight and λ is the adaptive strength coefficient.

The final total loss is

(15) $$L_{total}=L_{quantile}+\beta_{adaptive}\cdot L_{calib}$$

This adaptive mechanism ensures that when coverage deviation is large, the model automatically increases the weight of calibration loss, promoting rapid convergence of prediction intervals to target coverage.

#### 2.3.4. Closed-Loop Calibration (Feedback) Mechanism

The proposed framework incorporates an explicit coverage-feedback loop to maintain reliable prediction intervals under nonstationary flotation conditions. First, during training of the quantile regression heads, the empirical coverage of each target quantile is computed on mini-batches and treated as a feedback signal. To suppress batch-to-batch fluctuations, an exponential moving average (EMA) is used to track the smoothed coverage trajectory. The deviation between EMA coverage and the theoretical target coverage is then converted into an adaptive gain to dynamically reweight the calibration penalty term (i.e., a larger deviation leads to a larger $\beta_{\mathrm{adaptive}}$, thereby accelerating convergence of interval coverage while preserving point accuracy). This procedure corresponds to the EMA coverage update and adaptive weighting strategy in Equations (11)–(15) and is implemented in the training loss where EMA coverage is updated online and the calibration weight is increased when the coverage mismatch becomes large.

Second, at the interval construction stage, the localized conformal prediction module provides a second feedback loop: for each test sample, a local nonconformity radius is computed from its *k*-nearest calibration neighbors in the fused feature space, and a global width multiplier *m* is iteratively adjusted on the calibration set to meet the target coverage (Algorithm 1). In industrial deployment, this closed-loop calibration can be executed periodically using a sliding window of the most recent labeled samples (e.g., once per shift or every N assays), which enables continuous re-calibration under ore property drift and operating condition changes.
**Algorithm 1** Iterative optimization strategy**1**. Initialize width multiplier m = 1.2.**2**. Evaluate current coverage $C_{current}$ on calibration set.**3**. If $C_{current}<C_{target}$,Calculate the coverage gap: $\delta =C_{target}-C_{current}$.Update the Nonlinear multiplier: m←m×(1+3δ).Repeat steps **2–3** for maximum *T* iterations.The final prediction interval is$CI(x^{*})=[{\hat{y}}_{0.5}(x^{*})-m\cdot r(x^{*}),{\hat{y}}_{0.5}(x^{*})+m\cdot r(x^{*})]$.

### 2.4. Localized Conformal Prediction Module

While quantile regression can provide prediction intervals, it assumes uniform data distribution and struggles to adapt to local data heterogeneity. Therefore, we introduce localized conformal prediction methods that provide more precise prediction intervals by considering local neighborhood information of samples.

#### 2.4.1. Calculation of the Nonconformity Score

During calibration, for each sample $\left(x_{i},\;y_{i}\right)$ in the calibration set, its nonconformity score is calculated by,

(16) $$s_{i}=\left|y_{i}-{\hat{y}}_{0.5}\left(x_{i}\right)\right|$$

where ${\hat{y}}_{0.5}\left(x_{i}\right)$ is the median prediction. The corresponding feature vector $f_{i}\;=\;F_{fused}\left(x_{i}\right)$ is also stored.

#### 2.4.2. Construction of the Localized Prediction Interval

For test sample $x^{*}$, first, its features $f^{*}=F_{fused}\left(x^{*}\right)$ are extracted, then, Euclidean distances to all samples are calculated in the calibration set:

(17) $$d_{i}=\|f^{*}-f_{i}{\|}_{2},\;\;i=1,2,\ldots ,n_{cal}$$

Select k-nearest neighbors to obtain the local nonconformity score set as $S_{k}=\left\{s_{\left(1\right)},s_{\left(2\right)},\ldots ,s_{\left(k\right)}\right\}$.

Based on significance level α, the local calibration factor is calculated by,

(18) $$r\left(x^{*}\right)=Q_{1-\alpha }\left(S_{k}\right)$$

where $Q_{1-\alpha }(\cdot )$ represents the (1−α) quantile.

#### 2.4.3. Optimization of the Adaptive Interval Width

To ensure that the prediction intervals can achieve target coverage, an iterative optimization strategy is designed. The algorithm is as shown in Algorithm 1.

This local neighbor-based strategy provides statistically guaranteed prediction intervals tailored to the local data distribution of specific test samples.

### 2.5. End-to-End Training Strategy

We adopt an end-to-end training strategy which optimizes the feature extractors and the uncertainty prediction modules simultaneously. The training procedure consists of three stages:

**Stage 1: Feature Extractor Pre-training**

First, SKNet and ViT are pre-trained using standard mean squared error loss to learn capabilities of basic feature representation.

**Stage 2: Joint Training**

Feature extractors are then jointly trained with the adaptive quantile regression module using Adam optimizer with learning rate of 0.001 and cosine annealing scheduling.

**Stage 3: Localized Calibration**

Localized conformal calibration is performed on the validation set, determining optimal interval width multipliers through iterative optimization. The complete algorithm pseudocode is shown in Algorithm 2.
**Algorithm 2** Our method in this study **Feature Extraction:** For each image x,$f_{sk}$ = SKNet(x)$f_{vit}$= ViT(x)$f_{fused}$ = CrossModalFusion($f_{sk}$,$f_{vit}$) **Adaptive Quantile Regression Training:**Initialize model parameters θ**for** epoch in 1...N **do**  **for** (x_batch, y_batch) in D_train **do**     $y_{pred}$ = QuantileRegression($f_{fused}$,θ)     Compute $L_{quantile}$, $L_{calibration}$     Update Coverage_ema     $L_{total}=L_{quantile}+\beta_{adaptive}\cdot L_{calib}$     Backpropagate and update θ  **end for****end for** **Localized Conformal Calibration:**Extract features and nonconformity scores on calibration set.Optimize width factor m for target coverage.**Prediction:** For a new sample x_test:$y_{median}$ = Median prediction from model$r\left(x_{test}\right)$ = K-NN-based local calibration factor    $CI\left(x_{test}\right)=\left[y_{median}-m\cdot r\left(x_{test}\right),y_{median}+m\cdot r\left(x_{test}\right)\right]$**Return**$y_{median}$, $CI\left(x_{test}\right)$

## 3. Experimental Results and Analysis

### 3.1. Experimental Setup and Environment

In industrial flotation process, frequent shifts in ore properties, reagent dosages, and operating conditions make the process highly nonlinear and time varying. As a result, grade prediction models must deliver not only accurate point estimates but also reliable uncertainty to remain robust under noise, drift, and delays. Accurate point estimates are essential for process optimization, while reliable uncertainty quantification is critical for risk management, anomaly detection, and intelligent process control. Therefore, models must achieve both high prediction accuracy and robust, trustworthy uncertainty estimates to be applicable in real-world production environments.

#### 3.1.1. Image Acquisition Setup

**Figure 2:**
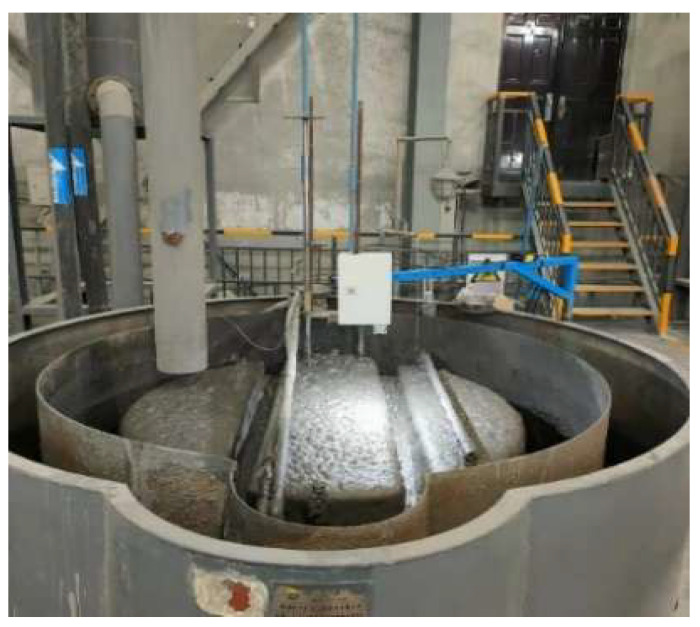
Schematic diagram of the flotation froth image acquisition device.

For the collection of froth images, a dedicated device was deployed in the plant’s production scene, as shown in Figure 2 (flotation froth picture acquisition device). Froth images were captured on-site using a Hikvision industrial camera mounted above the flotation column, pointing vertically downward to cover the region of interest on the froth surface. To ensure high-quality imaging, the camera was rigidly fixed to minimize vibration-induced motion blur, and a bar-shaped auxiliary light source was installed to provide stable illumination, thereby reducing the influence of ambient light variations. The camera operated in continuous acquisition mode, recording froth images in real-time at a fixed sampling interval.

#### 3.1.2. Label Alignment with Assay Measurements

**Figure 3:**
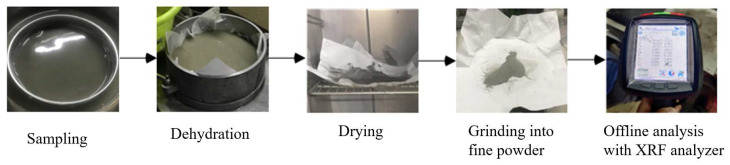
Flotation Froth Grade Assay Process.

For the acquisition of grade labels matching the froth samples, a standardized assay process was strictly executed—covering sampling, dehydration, drying, grinding into fine powder, and offline analysis with an XRF analyzer. The detailed workflow is presented in Figure 3 (flotation froth grade assay process). Concentrate grade labels were obtained from these offline assay measurements at approximately one-hour intervals. Since the assay label is inherently time-delayed relative to the instantaneous froth appearance, each label was aligned to the corresponding image samples by timestamp matching. Specifically, for each assay timestamp, the froth image closest in time was selected as the paired input to ensure temporal consistency between the visual features and the chemical analysis.

**Figure 4:**
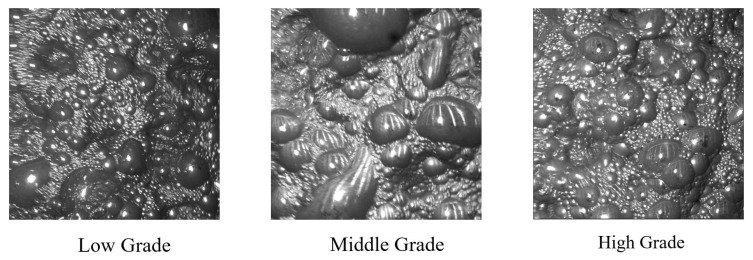
Representative froth images corresponding to different concentrate grades (low, medium, and high).

To further elucidate the data characteristics and the correlation between surface morphology and flotation performance, Figure 4 illustrates representative froth samples across different grade intervals. These visual variations are direct consequences of the fluctuating operating conditions (e.g., changes in air flow rate, pulp level, and reagent regime) inherent to the industrial process. As shown in the figure, the low-grade froth is characterized by large, irregular bubbles with a “watery” texture and poor stability, indicating low mineralization and significant gangue entrainment. Conversely, the high-grade froth manifests as a compact, fine-grained structure with uniform bubble size and a distinct luster, reflecting a high loading of hydrophobic valuable minerals and a stable froth layer. The middle-grade froth represents a transitional state with intermediate bubble size and viscosity. Incorporating such diverse visual patterns into the dataset is crucial for the model to learn robust feature representations that generalize well across the complex, nonlinear dynamics of the flotation process.

To validate the effectiveness and practicality of our proposed method, we conducted systematic experimental validation using real industrial data. The data was collected from a tungsten flotation plant from January to June 2022, comprising 1800 froth images with corresponding grade labels. The dataset was split into training, validation, and test sets in an 8:1:1 ratio.

The configuration of the experimental environment is,

**Hardware**: NVIDIA RTX 3090 GPU (24 GB memory), 32GB RAM, AMD 5800X CPU.

**Software**: PyTorch 2.41, Python 3.12, CUDA 12.4.

**Hyperparameters**: Learning rate 0.001, batch size 16, training epochs 100.

The backbone network structure parameters are set based on the input size and computing power constraints; uncertainties and conformal related hyperparameters (such as *α*, *k*, width multiplier, etc.) are determined on the validation set through grid search/sensitivity analysis, with the criterion of “minimizing the interval width and error while satisfying the target coverage”.

**The experimental** workflow includes the following:

**Data preprocessing:** Each froth image was loaded using Python Imaging Library (PIL) and converted to RGB to ensure a consistent three-channel input. Images were then resized to 600 × 600 and converted to tensors. Finally, channel-wise normalization was applied using mean [0.485, 0.456, 0.406] and standard deviation [0.229, 0.224, 0.225]. Unless otherwise specified, no additional data augmentation was applied, so that the learned representations reflect real industrial imaging conditions without introducing artificial geometric or photometric distortions.

**Model training:** A total of 80% of the data was used for training, 10% for validation, and 10% for testing. Both quantile and calibration losses were monitored.

**Performance evaluation:** On the test set, we report MAE, RMSE, and R^2^ for point accuracy, and assess uncertainty with prediction-interval coverage (PICP) and mean interval width (MIW), for both the model’s quantile-based intervals and the localized conformal intervals.

### 3.2. Evaluation Metrics

To comprehensively assess both regression performance and uncertainty quantification for prediction of grade in froth flotation, a two-dimensional evaluation system was established, encompassing regression accuracy and prediction interval metrics.

#### 3.2.1. Regression Accuracy Metrics

Regression accuracy metrics include Mean Absolute Error (MAE), Root Mean Squared Error (RMSE), and coefficient of determination (R^2^).

(19) $$MAE=\frac{1}{n}\sum_{i=1}^{n}\left|{\hat{y}}_{i}-y_{i}\right|\;$$

(20) $$RMSE=\sqrt{\frac{1}{n}\sum_{i=1}^{n}{\left({\hat{y}}_{i}-y_{i}\right)}^{2}}$$

(21) $$R^{2}=1-\frac{\sum_{i=1}^{n}{\left({\hat{y}}_{i}-y_{i}\right)}^{2}}{\sum_{i=1}^{n}{\left(y_{i}-\bar{y}\right)}^{2}}$$

where ${\hat{y}}_{i}$ and $y_{i}$ are the predicted and true values of the *i*-th sample, respectively, and $\bar{y}$ is the mean of all the true values.

#### 3.2.2. Prediction Interval Metrics

For quantifying model uncertainty characterization, the method was further evaluated from the perspective of prediction intervals by using the following metrics.

**Coverage**: Proportion of true values contained within the predicted intervals.

**Mean Interval Width** (MIW): Average width of the prediction intervals.

(22) $$Coverage=\frac{1}{n}\sum_{i=1}^{n}I\left(y_{i}\in [L_{i},U_{i}]\right)$$

(23) $$MIW=\frac{1}{n}\sum_{i=1}^{n}\left(U_{i}-L_{i}\right)$$

where $L_{i}$ and $U_{i}$ are the lower and upper bounds of the prediction interval for the *i*-th sample, and I(.) is the indicator function.

For LCP, *k*-nearest neighbor statistics were used to compute personalized intervals, and the same metrics were applied.

### 3.3. Baseline Model Design

A robust and representative baseline is essential for objectively evaluating the contributions of the proposed method. The baseline combined state-of-the-art components without integrating the core innovations introduced in this work. Specifically, it used a multi-modal feature extraction and quantile regression framework as follows.

At the feature extraction level, we construct a SKNet-ViT Fusion network combining SKNet’s convolutional attention mechanism with ViT’s self-attention mechanism. The SKNet branch captures local spatial features through selective kernel convolution, and the ViT branch extracts global semantic information through transformer blocks, where each branch outputs 128-dimensional feature vectors. These feature vectors are concatenated to form a 256-dimensional vector, which is then fused through a two-layer fully connected network (256 → 256 → 256). For uncertainty modeling, a standard quantile regression method is employed. Independent prediction heads are constructed for three preset quantiles (0.05, 0.5, 0.95). Each head consists of three fully connected layers and is trained with the standard quantile (pinball) loss.

**Figure 5:**
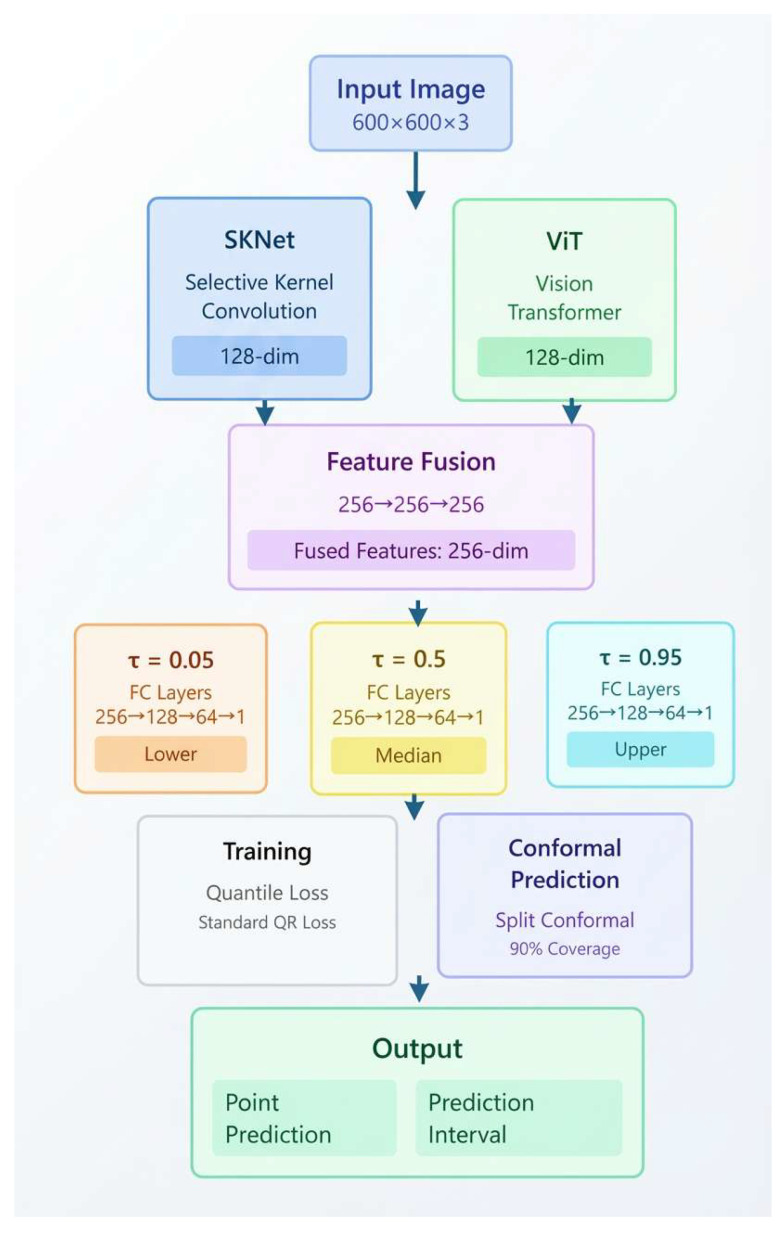
Baseline Model Architecture.

For prediction interval construction, basic split conformal prediction method is implemented by using absolute errors of median predictions on the validation set as nonconformity scores. We construct prediction intervals with 90% coverage by applying statistical calibration. The baseline SKNet–ViT fusion architecture and the corresponding uncertainty estimation pipeline are shown in Figure 5.

**Figure 6:**
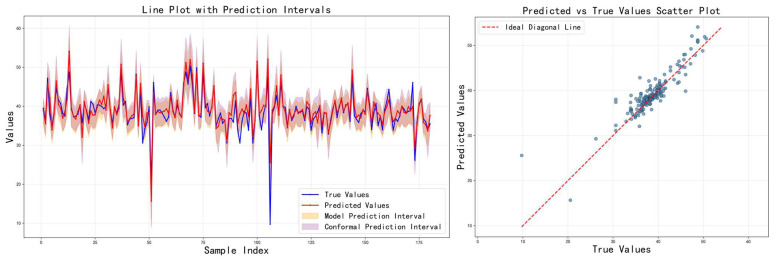
Prediction results of Baseline method.

Figure 6 shows the prediction results of the baseline method on the test set, including comparison curves between predicted values and actual values and coverage of prediction intervals. It can be observed that while the method can reasonably match the true grades in some samples, the prediction intervals are often too wide or too narrow, with significant fluctuations in coverage and interval width. This indicates that the standard quantile regression and global conformal methods have significant limitations in complex scenarios. This means that improvement to the calibration and conformal prediction is necessary.

### 3.4. Results and Discussion

Building on the unified baseline, we adopt a three-step incremental optimization that targets the key modules without re-stating architectural details: (1) feature fusion, where progressively more interactive schemes are compared and the cross-modal interaction variant is selected; (2) uncertainty loss design, where formulations that promote calibration and quantile ordering are assessed and the EMA-based adaptive calibration is chosen; and (3) interval construction, where personalized conformal variants are evaluated and localized conformal prediction is adopted. Each ablation swaps a single component while keeping all other settings fixed and is evaluated with MAE/RMSE/R^2^ and interval metrics (PICP/MIW). The best choices from the three ablations are then integrated into the final model, yielding consistent gains over the baseline in both point accuracy and calibrated sharpness.

Table 1 lists the parameter settings for each main model in detail, including SKNet’s input size, convolution kernel size, branch parameters, and feature channel count, as well as ViT’s patch size, embedding dimension, multi-head attention count, number of layers, and so on. A clear parameter table lays the foundation for method reproducibility and fair comparison, while also reflecting the trade-offs between model scale and complexity.

Table 2 provides a detailed overview of the uncertainty modeling strategies employed by each comparison method, including quantile settings, network head structures, loss function forms, and key parameters. This table clearly illustrates the similarities and differences between the methods in terms of uncertainty quantification design for experimental comparisons.

#### 3.4.1. Feature Fusion Strategies

Feature quality is a key factor in determining the upper limit of model performance. SKNet excels at capturing local texture details, while ViT focuses on global structure modeling. Effectively integrating these two complementary feature representations is the primary issue to be addressed. This section compares three different fusion strategies: attention-weighted fusion learns adaptive weights to balance the contributions of different features, cross-modal interaction fusion enhances feature expression through bidirectional information exchange, and adaptive feature selection fusion uses a gating mechanism to perform content-aware feature selection.

Three fusion mechanisms were compared:

(1). Attention-weighted fusion: Adaptive attention weights are learned to balance the contributions of SKNet and ViT features, dynamically assigning more weight to the most relevant features per sample.
(2). Cross-modal interaction fusion: bidirectional attention modules are employed to facilitate mutual guidance and deep feature interaction between SKNet and ViT, enhancing expressiveness and complementarity.
(3). Adaptive feature selection fusion: a gating mechanism is used to adaptively select and combine features at a granular level, complemented by residual connections to preserve feature diversity.

Cross-modal interaction fusion achieved the best trade-off between regression accuracy and prediction interval metrics, highlighting the value of deep bidirectional feature integration.

**Figure 7:**
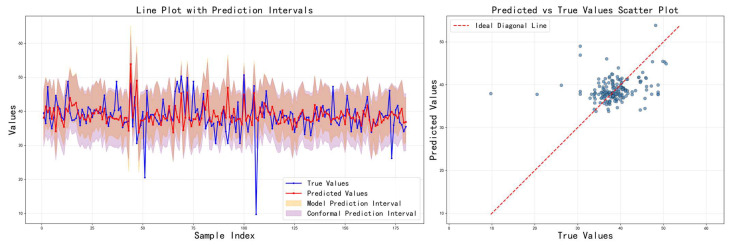
Prediction results on the test set using the attention-weighted fusion strategy.

**Figure 8:**
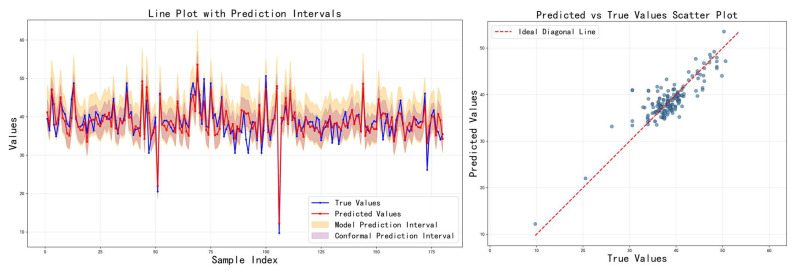
Prediction results on the test set using the cross-modal interaction fusion strategy.

**Figure 9:**
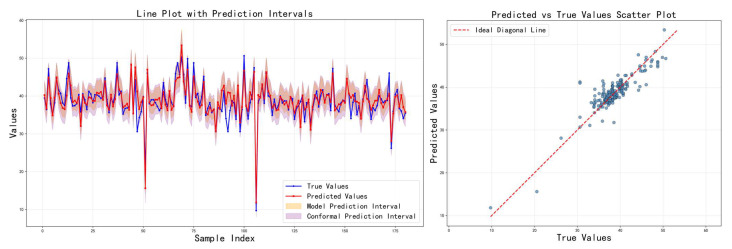
Prediction results on the test set using the adaptive feature selection fusion strategy.

Table 3 shows the comparison results of the three feature fusion strategies in terms of various indicators, and the corresponding prediction results are visualized in Figure 7, Figure 8 and Figure 9, respectively. It can be seen that cross-modal interaction fusion outperforms the other two methods in terms of accuracy indicators such as MAE, RMSE, and R^2^, and also performs more evenly in terms of interval coverage and width. This result verifies that the bidirectional attention mechanism can effectively improve feature expression capabilities, further supporting the rationality of the design of the method in this paper.

#### 3.4.2. Uncertainty Modeling with Multiple Loss Functions

Accurate quantification of uncertainty is a core requirement for industrial applications. While traditional quantile regression can provide prediction intervals, it often suffers from issues such as unstable coverage and overlapping intervals. This section explores three improved loss function designs: the hybrid uncertainty model combines the advantages of quantile and distribution modeling, the ranking consistency model ensures the reasonableness of prediction intervals, and the adaptive calibration model achieves precise control of coverage through dynamic adjustment. These methods improve the quality of uncertainty modeling from different perspectives.

Three advanced uncertainty modeling strategies were explored:

(1). Hybrid Uncertainty Model (HUM)

The HUM combines quantile regression with Gaussian distribution modeling. It optimizes a composite objective that balances quantile loss, negative log-likelihood, and a consistency loss, thereby aligning quantile-based and distributional predictions.

(2). Rank Consistency Model (RCM)

The RCM enforces monotonicity among predicted quantiles. By ensuring that larger prediction errors correspond to wider intervals, it prevents quantile crossing and maintains coherent interval ordering.

(3). Adaptive Calibration Model (ACM)

The ACM dynamically adjusts calibration weights using an exponential moving average (EMA) of coverage rates. This mechanism steers the model toward the target coverage level without manual hyperparameter tuning.

**Figure 10:**
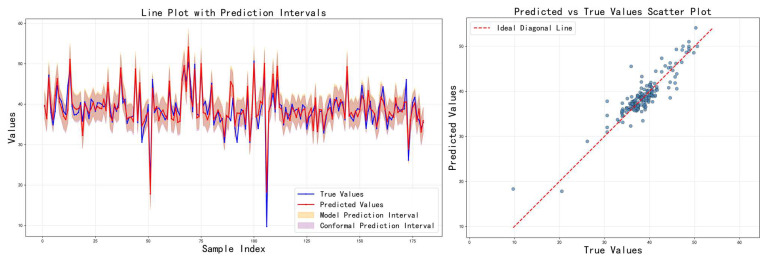
Prediction results on the test set using the hybrid uncertainty model.

**Figure 11:**
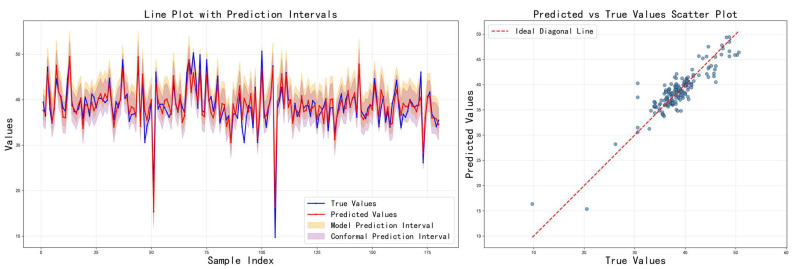
Prediction results on the test set using the rank consistency model.

**Figure 12:**
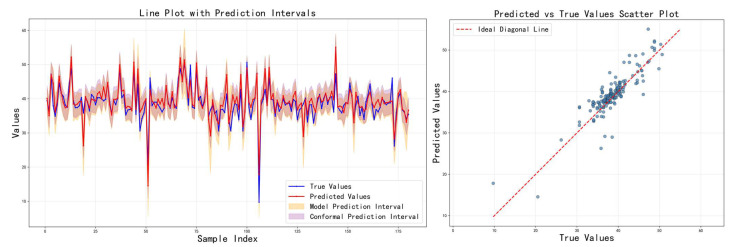
Prediction results on the test set using the adaptive calibration model.

Table 4 shows the impact of different loss functions on the model performance. The adaptive calibration model performs best, achieving the narrowest model prediction interval while maintaining high prediction accuracy. Although its model coverage appears low, this precisely highlights the method’s advantage: it shifts the coverage guarantee to the subsequent conformal prediction stage, thereby avoiding overly conservative interval estimates. The key innovations are the following: (1) the EMA mechanism smooths coverage fluctuations between batches, providing a stable calibration signal; (2) adaptive weights are dynamically adjusted based on deviation, accelerating convergence; and (3) the optimization objectives of point prediction accuracy and interval coverage are separated. In contrast, the mixed uncertainty model suffers from performance trade-offs due to attempting to optimize multiple objectives simultaneously; while ordering consistency addresses interval overlap issues, it fails to effectively control coverage. The training curves in Figure 10, Figure 11 and Figure 12 show that the adaptive calibration model exhibits more stable loss convergence, with coverage gradually approaching the target value.

#### 3.4.3. Improved Conformal Prediction Methods

Conformal prediction provides prediction intervals with theoretical guarantees, but traditional global calibration methods ignore the local characteristics of the data. In the flotation process, the difficulty of prediction varies significantly under different operating conditions, and a unified calibration strategy is difficult to adapt to this heterogeneity. This section compares three personalized conformal prediction methods: conditional CP achieves adaptive interval adjustment through error prediction, quantile regression CP fully utilizes existing quantile information, and localized CP performs fine-tuned calibration based on similarity in the feature space.

Traditional global conformal calibration may fail under non-uniform or complex data distributions. To address this, we evaluate three personalized conformal prediction methods:

(1). Conditional Conformal Prediction (CCP)

CCP trains an auxiliary model to estimate the sample-wise expected absolute error and adjusts the prediction interval width for each instance accordingly, enabling individualized interval allocation.

(2). Quantile Regression Conformal Prediction (QR-CP)

QR-CP exploits quantile forecasts to calibrate the lower and upper bounds separately, improving interval sharpness and coverage, particularly for asymmetric target distributions.

(3). Localized Conformal Prediction (LCP)

LCP performs k-nearest-neighbor-based local calibration: for each test instance, it uses only the most similar calibration samples in the feature space, allowing the intervals to adapt to local data complexity. The results of three different conformal prediction methods are shown in Table 5. In terms of metrics, localized CP achieves a better balance between interval width and coverage, especially when dealing with complex, non-uniform data distributions. This shows that feature-space-based localized calibration can provide more personalized and reliable prediction intervals for each test sample, which is an effective means of improving practicality in industrial scenarios.

It is important to note that the regression accuracy metrics remain identical across all three methods in Table 5. This uniformity arises because conformal prediction functions as a post hoc calibration technique applied to the same underlying pre-trained base regressor. Consequently, while the point predictions and their associated errors remain unchanged, the distinct calibration strategies result in significant variations in interval widths and coverage rates.

Among the evaluated strategies, localized CP achieves the most favorable balance, yielding a reasonable mean interval width of 7.942 while maintaining a robust coverage rate of 92.2%. This superior performance is primarily attributed to the k-nearest neighbor strategy, which identifies similar samples in the feature space to provide customized calibration for each test point. Unlike global methods, these local statistics more accurately reflect the prediction difficulty inherent to specific regions of the data distribution. Conversely, although the quantile regression CP produces the narrowest intervals of 6.336, its coverage rate of 85.1% falls short of the theoretical 90% target, a deficiency likely caused by the bias inherent in direct quantile estimation. Similarly, while conditional CP offers theoretical flexibility, the requirement for an additional error prediction model introduces further uncertainty, leading to suboptimal performance compared to the localized approach. These findings collectively validate the effectiveness of personalized calibration rooted in local information.

Although the localized conformal prediction module involves k-nearest neighbor search, its computational overhead is fully controllable. The module consists of two phases: (1) the calibration phase is a one-time offline process that takes about 10 s, and (2) the online prediction phase, in which reasoning on a single image takes about 50–55 ms, with the k-nearest-neighbor search (180 × 256 distance calculations and sorting) accounting for only 3 ms. Considering that the time interval for grade prediction in real industrial applications is at least 3 min, the reasoning time is very low and has rich time surplus. The memory footprint of the module is about 50–55 MB, of which only a few hundred KB are needed for calibration data. k-nearest neighbor search is therefore not a system performance bottleneck, and the proposed method has good industrial deployability.

After module-wise validation, we assembled the final system using cross-modal interaction for feature extraction, adaptive calibration for uncertainty estimation, and localized conformal prediction for interval construction. We subsequently assessed performance with a suite of quantitative metrics and complementary visual analyses. To validate the effectiveness of the proposed method, this paper selects three mainstream uncertainty quantification methods for comparative experiments. MC Dropout maintains activated Dropout layers during inference and performs multiple stochastic forward passes, utilizing the mean and standard deviation of prediction results to estimate epistemic uncertainty, with prediction intervals constructed through quantiles of the standard normal distribution. Deep Ensemble employs an ensemble model composed of multiple independently trained neural networks, where each member network simultaneously outputs prediction means and variance, quantifying model uncertainty by ensembling the predictive distributions of all members, with the total variance comprising the mean of member prediction variances (aleatoric uncertainty) and the variance of member prediction means (epistemic uncertainty). DKL-GP (Deep Kernel Learning with Gaussian Process) combines the feature extraction capability of deep learning with the uncertainty modeling advantages of Gaussian processes, mapping inputs to a latent space through neural networks and then leveraging local neighborhood information and residual statistics of training samples to estimate predictive uncertainty, with prediction intervals comprehensively considering base variance, distance uncertainty, and neighborhood residual standard deviation. These three methods, respectively, represent uncertainty quantification paradigms based on stochastic inference, model ensembling, and kernel methods, providing comprehensive comparison benchmarks for the proposed method.

**Figure 13:**
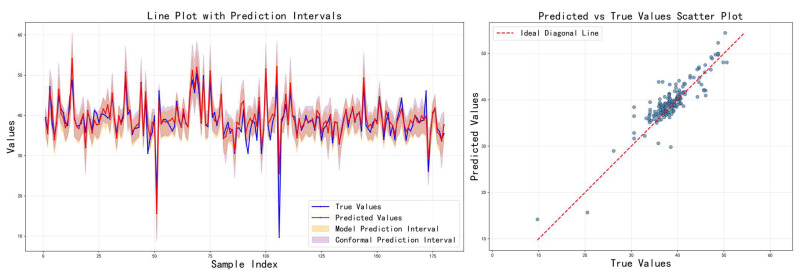
Prediction results of proposed method.

As shown in Figure 13 and Table 6, experimental results show that the proposed method not only achieves low error in point predictions but also effectively covers most real observations with its prediction intervals, demonstrating good convergence in interval width, thereby intuitively showcasing the model’s reliability and practicality. Specifically, cross-modal fusion fully exploits local and global information, the adaptive calibration mechanism effectively controls the coverage of prediction intervals, and localized conformal prediction targets personalized feature distributions to enhance the accuracy of confidence intervals. Overall, the proposed method performs exceptionally well in quantifying uncertainty, providing robust data support and reference for industrial decision-making.

## 4. Conclusions

In this paper, we proposed a novel method for flotation grade prediction with uncertainty quantification, based on cross-modal feature fusion and adaptive calibration. By designing a network that fuses multi-scale information from froth images, an adaptive mechanism that ensures accurate interval coverage, and a localized conformal prediction module that provides theoretically guaranteed uncertainty estimates, our method effectively addresses key challenges in industrial process modeling.

Overall, our method demonstrates significant potential in fusing multi-modal information, performing robust uncertainty quantification, and generating localized predictions. It offers new insights and a powerful framework for regression and interval estimation tasks in complex industrial processes.

Future work will focus on incorporating temporal information to better handle process drifts and exploring semi-supervised learning to leverage unlabeled data, further enhancing the model’s adaptability in dynamic industrial environments.

## Figures and Tables

**Table 1:** Detailed parameter settings for the SKNet and ViT backbone networks.

Model	Parameter Settings	
SKNet	Input size of image = 600 × 600 Kernel sizes = [7 × 7, 5 × 5, 3 × 3] SK parameters: M = 2, G = 32, r = 16, L = 32	Channels = 3 Feature channels = [64, 128, 256]
ViT	Input size of image = 600 × 600 Embed dimension = 128 Attention heads = 8 Dropout rates = 0.1	Patch size = 50 × 50 Layers = 6 MLP ratio = 4

**Table 2:** Comparison of different uncertainty quantification methods and their configurations.

Method	Network Structure	Loss Parameters
Baseline QR	3 × [FC (256 → 128 → 64 → 1)]	Standard quantile loss
Hybrid Uncertainty	Quantile + Gaussian heads	α = 0.7, λ = 0.3
Rank Consistency	3 × [FC (256 → 128 → 64 → 1)]	γ = 0.5, margin = 0.1
Adaptive Calibration	3 × [FC (256 → 128 → 64 → 1)]	β = 0.5, momentum = 0.9

**Table 3:** Comparison of experimental results under different feature fusion strategies.

Method	MAE	MSE	RMSE	R^2^	Model Coverage	Model Interval Width	CP Coverage	CP Interval Width
Attention Weighted	1.620	5.381	2.320	0.758	0.922	7.661	0.911	8.011
Cross-Modal Interaction	1.562	4.973	2.243	0.773	0.928	6.976	0.939	6.446
Adaptive Feature Selection	2.042	6.950	2.636	0.687	0.861	8.077	0.839	7.163

**Table 4:** Comparison of experimental results under different loss functions.

Method	MAE	MSE	RMSE	R^2^	Model Coverage	Model Interval Width	CP Coverage	CP Interval Width
Mixed Uncertainty	2.250	8.941	2.990	0.597	0.856	6.366	0.85	7.535
Sorting Consistency	1.671	6.107	2.471	0.725	0.867	6.061	0.917	7.594
Adaptive Calibration	1.613	5.277	2.268	0.766	0.806	5.589	0.911	7.582

**Table 5:** Experimental results by using different conformal prediction methods.

Method	MAE	MSE	RMSE	R^2^	Model Coverage	Model Interval Width	CP Coverage	CP Interval Width
Conditional CP	1.858	7.793	2.792	0.633	0.844	6.278	0.910	7.644
Quantile Regression CP	1.858	7.793	2.792	0.633	0.844	6.278	0.851	6.336
Localized CP	1.858	7.793	2.792	0.633	0.844	6.278	0.922	7.942

**Table 6:** Performance comparison between the proposed method and other approaches.

Method	MAE	MSE	RMSE	R^2^	Model Coverage	Model Interval Width	CP Coverage	CP Interval Width
MC Dropout	2.3640	10.596	3.255	0.656	0.933	13.827		
Deep Ensemble	2.3767	9.105	3.017	0.705	0.933	11.22		
DKL-GP	2.2355	9.062	3.010	0.706	0.95	14.179		
Proposed method	1.358	4.811	2.193	0.783	0.955	6.827	0.961	7.779

## Data Availability

No new data were created or analyzed in this study.

## Appendix A. Mathematical Details of Feature Extractors

### Appendix A.1. Multi-Scale Local Feature Extraction with SKNet

SKNet enhances a model’s ability to capture information at different spatial scales through an adaptive combination of convolutional kernels [32]. To extract the rich, local, multi-scale features from flotation froth images, we employ the Selective Kernel (SK) module. The SK module processes the input feature map through multiple parallel convolutional branches, with a different dilation rate and kernel size for each branch. It then uses global information to adaptively assign weights to each branch.

For input flotation froth image $x\in R^{C\times H\times W}$, SKNet first extracts features at different scales through *M* parallel branches:

(A1) $$F_{i}\left(x\right)=\mathrm{ReLU}(\mathrm{BN}({Conv}_{d_{i}}(x)))$$

where Conv_*di*_(⋅) is a convolution operation with a specific dilation rate $d_{i}$, denotes batch normalization, and ReLU is the activation function.

Different dilation rates provide branches with different receptive fields, capturing multi-scale information.

Subsequently, unified representation is obtained through feature aggregation:

(A2) $$F_{U}\left(x\right)=\sum_{i=1}^{M}F_{i}\left(x\right)$$

To achieve adaptive feature selection, SKNet generates attention weights from global information. First, Global Average Pooling (GAP) is used to compress spatial information:

(A3) $$Z=GAP\left(F_{U}\right)=\frac{1}{H\times W}\sum_{h=1}^{H}\sum_{w=1}^{W}F_{U}\left(h,w\right)$$

Next, a two-layer fully connected network generates channel-wise attention weights:

(A4) s=FC2(ReLU(FC1(z)))

where FC_1_ maps the GAP vector $s\in R^{c}$ to a lower-dimensional vector $z\in R^{d}$, followed by a pointwise nonlinearity (e.g., ReLU). FC_2_ expands $z\in R^{d}$ to a higher-dimensional vector $a\in R^{M\times C}$, which is then reshaped into a matrix $A\in R^{M\times C}.d=\max\left(\frac{C}{r},L\right)$ is the reduced feature dimension, r is the reduction ratio, and L is the minimum dimension threshold.

The selection weights for each branch are computed using a softmax function:

(A5) $$w_{i}=\frac{\exp(s_{i})}{\sum_{j=1}^{M}exp{(s}_{j})}$$

Finally, the output of the SKNet module is a weighted sum of the features from each branch:

(A6) $$F_{SK}\left(x\right)=\sum_{i=1}^{M}w_{i}⊙F_{i}\left(x\right)$$

where ⊙ denotes element-wise multiplication.

This adaptive mechanism enables the network to dynamically adjust the importance of features at different scales based on input content.

### Appendix A.2. Global Feature Extraction with ViT

To capture global dependencies and long-range interactions within the froth images, we introduce a ViT as a global feature extractor. ViT models the relationships between image patches using a self-attention mechanism, effectively complementing the local focus of SKNet.

#### Appendix A.2.1. Image Patch Embedding and Position Encoding

Given input image $X\in R^{H\times W\times 3}$, it is first divided into non-overlapping patches. With patch size p×p, the image is divided into $N=\frac{HW}{p^{2}}$ patches. Each patch is mapped to *d*-dimensional embedding space through linear projection:

(A7) $$z_{0}=\left[x_{cls};x_{p}^{1}E;x_{p}^{2}E;\ldots ;x_{p}^{N}E\right]+E_{pos}$$

where $x_{\mathrm{cls}}\in R^{d}$ is a learnable classification token, $E\in R^{p^{2}\cdot 3\times d}$ is the patch embedding matrix, and $E_{\mathrm{pos}}\in R^{\left(N+1\right)\times d}$ is position encoding.

#### Appendix A.2.2. Multi-Head Self-Attention Mechanism

The core of the transformer encoder is the multi-head self-attention (MHSA) mechanism [33]. For input sequence $Z\in R^{\left(N+1\right)\times d}$, MHSA is calculated as:

(A8) $$Attention\left(Q,K,V\right)=soft\max\left(\frac{QK^{T}}{\sqrt{d_{k}}}\right)V$$

where query matrix $Q=ZW_{Q}$, key matrix $K=ZW_{K}$, value matrix $V=ZW_{V}$, and $d_{k}$ is the dimension per attention head.

Multi-head attention captures different representation subspaces by computing h attention heads in parallel:

(A9) $$MHSA\left(Z\right)=Concat\left(head_{1},\ldots ,head_{h}\right)W_{O}$$

where $head_{i}=Attention\left(ZW_{Q}^{i},ZW_{K}^{i},ZW_{V}^{i}\right)$.

#### Appendix A.2.3. Transformer Encoder Block

Each transformer block contains MHSA and feed-forward network (FFN), enhanced with residual connections and layer normalization for training stability:

(A10) $$Z^{’}=Z+MHSA\left(LN\left(Z\right)\right)\;Z^{\prime \prime }=Z^{’}+FFN\left(LN\left(Z^{’}\right)\right)$$

where LN(⋅) denotes layer normalization, and FFN(⋅) is a two-layer fully connected network which is,

(A11) $$FFN\left(x\right)=GELU\left(W_{1}x+b_{1}\right)W_{2}+b_{2}$$

where GELU denotes the Gaussian Error Linear Unit activation function.

After L transformer blocks, the feature corresponding to the classification token is extracted as global representation:

(A12) $$F_{ViT}=Z_{L}^{0}$$
